# How does negative new media coverage impact audit fees, cost cover, or risk premium? Based on the data from WeChat official account by Crawler Technology

**DOI:** 10.1371/journal.pone.0297237

**Published:** 2025-02-26

**Authors:** Tao Feng, Chuan Zhang, LiXia Liu, Xin Lin

**Affiliations:** 1 School of Economics and Management, Shanghai Maritime University, Shanghai, China; 2 Shanghai University of Electric Power, Shanghai, China; 3 Shanghai Dianji University, Shanghai, China; 4 Shanghai Haishun New Pharmaceutical Packaging Co., Ltd, Shanghai, China; University of Malta, MALTA

## Abstract

This study empirically investigates the relationship between negative new media coverage and audit fees by collecting a sample of nonfinancial listed companies on the main board of the Shanghai Stock Exchange (SSE) from 2017 to 2019, along with data on negative new media coverage of official WeChat public accounts founded by the most influential national financial newspaper obtained using a crawler. This study revealed that: 1. Audit fees are significantly and positively related to negative news media coverage; 2. The increase in audit fees is due to the fact that if a listed company undergoes negative new media coverage, the accounting firm that audits the company will consequently increase risk premium and thus audit fees. We found no evidence that accounting firms spend more time and effort on audit work after a listed company experiences negative new media coverage; 3. Analyst-tracking has a moderating effect on negative new media coverage and audit fees, leading to an increase in audit fees; 4. Negative media reporting on non-state-owned enterprises is more likely to cause accounting firms to increase their audit fees than their state-owned peers, whereas “non-Big Four” accounting firms are more likely to increase their audit fees for companies with negative new media reports. This study, based on the differing impacts of negative new media coverage, intended to unravel the intricate relationship between different types of negative new media coverage and audit fees, help understand the mechanism whereby negative reporting impacts audit fees, and provides a benchmark for the development of a feasible audit fee system.

## 1. Introduction

In the tidal wave of the Information Age, new media are playing increasingly important roles in the development of China’s social economy. In particular, their roles in exposing the financial fraud of listed companies like “Roe deer Island,” “Beidahuang,” “Fujian Jinsen,” and “Shandong Molong” have been widely observed. New media is a relatively broad concept, typically referring to the emergence since the early 21st century of providing information and services to users through digital, interactive multimedia terminals (Fan et al., 2022) [1]. This contrasts with traditional media, such as print, radio, and television. Most new media are disseminated via the internet, allowing for global, real-time access, and sharing. Many new media platforms offer instant messaging features, enabling users to share and receive information in real time. New media is an information medium that uses the internet as a carrier and applies digital technology for production & dissemination. The core difference between new media and traditional media is the organized participation of the public and the internet as the information carrier (Kane et al., 2014) [2]. New media allows the public to share information and contribute ideas via the internet, breaking the authority and one-way nature of traditional media (Cade, 2018), and has had significant economic implications for the capital market [3].

With the rapid development of digital technology, the timeliness of new media information dissemination has been greatly enhanced, and WeChat has become one of the most influential new media platforms in the daily life and work of the public. Users browsing information and posting comments on new media online platforms have promoted a more balanced and diversified expression of public opinion, enhancing the overall transparency of information in society. This has, in turn, created a more open and transparent environment for information disclosure. New media is the primary force driving online public sentiment. In recent years, there have been many studies on the formation, evolution, early warning, and governance strategies of new media public sentiment. However, there has been less research on the mechanisms by which new media information affects the decision-making behaviors of specific industry groups and the extent of its impact.

The auditor, acting as one of the supervisors in the capital market, plays a pivotal role in safeguarding the high-quality development of the economy and society. Audit fees, representing the auditor’s direct perception of a client’s risk profile, are one of the main focuses of audit research. Existing studies, such as those by Liang and Li (2022), have extensively examined the impact of company characteristics and the traits of accounting firms on the audit fees of publicly-listed companies [4]. Beyond that, the influence of new media, analysts, and other external information intermediaries on corporate operations and management has started to attract wide attention from scholars, as seen in research by Yi and others (2019) [5].

With the rapid advancement of internet technology, the prevalent modes of information distribution have witnessed significant transformations, making new media an integral medium of information dissemination. As highlighted by the “51st China Internet Development Statistics Report” released by the China Internet Network Information Center in March 2023, by June 2022, China’s netizen population had surged to 1.067 billion, with an internet penetration rate of 75.6%. This widespread internet coverage has facilitated the growth of new media platforms, like WeChat official accounts. Consequently, information dissemination in the capital market has transitioned from a “one-to-many” model to a “many-to-many” paradigm, altering how investors access and interpret information. This has led to a reduction in the cost of acquiring information for investors and has enhanced the efficiency of information flow in the capital market. In the Report of the 20th CPC National Congress, the Chinese government explicitly stated: “To strengthen the building of the all-media communication system, shape a new mainstream public opinion landscape, perfect the comprehensive internet governance mechanism, and promote the establishment of a positive online environment.”

Given this context, investigating the impact of new media attention on audit fees holds significant theoretical and practical implications. Hence, can the disclosure of negative news about fraud by listed companies on new media platforms draw auditor attention and alert them to risks? How does the disclosure of negative news about fraud in listed companies on new media platforms affect auditors’ decisions regarding auditing practices?

Thus, this study empirically analyzes the relationship among negative new media coverage, analyst tracking, audit fees, and audit time lags for listed companies in China. Through the study, we found that:(1) There is a significant positive correlation between audit fees and negative news reports in new media. (2) The reason for the increase in audit fees is that when a publicly-listed company experiences negative coverage in new media, accounting firms subsequently raise their risk premium, leading to higher audit charges. We did not find evidence suggesting that accounting firms increase their audit working hours and efforts after such negative reports about listed companies emerge in new media. (3) The tracking by analysts moderates the relationship between negative news reports in new media and audit fees, leading to an increase in audit fees. (4) Non-state-owned enterprises that encounter negative news reports in new media are more likely to experience elevated audit fees from accounting firms. Furthermore, accounting firms outside the “Big Four” are more inclined to raise audit fees for enterprises that have been the subject of negative news reports in new media. Lastly, we conducted a categorized study on the different consequences of negative news reports in new media and sorted out the relationship between various types of negative news and audit fees. This research fills the gap regarding the impact and mechanism of new media’s online public sentiment on the micro-level audit fees of enterprises and provides some theoretical basis for the external supervisory effect of new media on corporate governance.

The main contributions of this paper are as follows: First, although some scholars have studied the relationship between news media attention and audit pricing in the past, their media data mainly come from the newspaper database of the China National Knowledge Infrastructure (CNKI). Alternatively, we argue that new media, represented by WeChat, spread quickly, and has a wide reach, allowing users to quickly access negative news about companies. In this study, the data on negative news were obtained from the official public accounts of WeChat media rather than from previous paper reports as research subjects. As the amount of data was too large to be collected manually, this study obtained it by utilizing the web crawler approach. Second, we conducted a multidimensional analysis of negative new media coverage, not only to explore the impact of negative new media coverage with varying consequences on audit costs but also to verify and analyze the paths and mechanisms of their impact in depth.

## 2. Literature review and research hypothesis

### 2.1 Negative media coverage and audit fees

Studies on the influence of media on the information decision-making behavior of market participants mainly involve the following two theoretical explanations: asymmetric information theory and stakeholder theory.

Asymmetric information theory believes that the theoretical basis for the media to play a governance effect lies in the phenomenon of information asymmetry between the two parties in market transactions. New media, through functions such as publishing, packaging, or transmitting, realizes the disclosure and in-depth dissemination of internal company information, alleviating the information asymmetry between it and the audited units, and constituting certain external pressure on audit risks. On one hand, auditors, when evaluating a company’s business risks, will consider all factors that may affect the company’s financial statements. When a company faces negative public opinion, it may lead to an increase in its operational risks, necessitating auditors to perform more in-depth and detailed audit work (Mo et al., 2022) [6]. Negative news may expose problems in some of the company’s management or operations. Auditors may need to reevaluate and test the company’s internal control systems. Auditors need to communicate more with the company management to obtain more information and explanations. In addition, if negative news involves possible litigation or claims (Sun et al., 2020), auditors need to evaluate these potential liabilities, all of which will increase the workload and complexity of the audit [7]. On the other hand, as a third-party independent institution, auditors may face greater public pressure to ensure the quality and credibility of their audit work when confronted with a company’s negative public opinion. This may lead auditors to adopt more cautious and strict auditing methods. In some cases, due to the negative news of the company, auditors may need to add additional explanations or notes in their audit reports (Wu et al., 2022) [8]. In summary, the negative public opinion faced by a company may increase the complexity, risk, and workload of the audit, leading to an increase in audit fees.

The stakeholder theory is a framework that emphasizes the interactions and relationships between a business and its various stakeholders. Stakeholders can be individuals or groups who have an interest in or are influenced by the business. Auditing firms are also stakeholders of the audited entity. Auditing firms have the responsibility to audit the financial reports of their clients accurately and fairly. Negative reports in the new media can indicate higher operational risks or potential financial issues for client companies. Therefore, for auditing firms, auditing such companies entails higher risks, which may lead to an increase in audit fees to compensate for the additional risk they undertake. Auditing firms are also highly concerned about their industry reputation, as it can impact their ability to acquire new clients and retain existing ones. If the entities they are auditing are companies that have been widely covered in negative news by the new media, auditing firms may need to conduct more rigorous audits to mitigate potential reputation risks. As a result, audit fees may also increase accordingly (Zhang and Yu, 2013) [33]. From a stakeholder perspective, auditing firms, as members of this framework, adjust the prices of their audit services based on the risks and responsibilities they face in dealing with various parties such as the business, investors, regulatory agencies, and the public.

#### 2.1.1 Negative reports of traditional media and company audit.

Many scholars have used collected data to find that negative traditional media coverage leads to increased audit fees. In the early years, scholars such as Mutchler et al. (1997) and Joe (2003) found a positive relationship between traditional media coverage and auditors’ opinions, as well as audit fees, notwithstanding that the incidents covered by the media are already disclosed to auditors [9,10]. Using Chinese A-share listed companies as the research sample, Ran and He (2014) suggested that the higher the media attention paid to a company, the higher its annual audit fees [11]. Meanwhile, Liu et al., (2014) found that in an environment of high litigation risk, negative media coverage in accounting would raise audit fees [12]. In addition, Hribar et al. (2014) found that the transformation of accounting firms would render auditors more sensitive to risk perception, thereby raising the risk premium component of audit fees [13]. Xiao and Zhang (2016) also studied the impact of negative media coverage on audit fees from the perspective of firms’ organizational change and found that negative company coverage and audit fees are significantly and positively correlated after transformation, but not before [14]. Mi et al. (2019) found that non-compliance with information disclosure increases audit fees by damaging the reputation of the involved firms and making them more likely to be blacklisted for future regulation by the regulator [15].

#### 2.1.2 New media and corporate governance.

In recent years, an increasing number of studies have examined the impact of new media, and scholars have verified the characteristics of new media coverage and its impact on enterprises. Huang (2015) pointed out in the People’s Daily in 2015 that the rapid development of new media has significantly improved the efficiency of social information dissemination compared with traditional media, insofar as its instantaneity and interactivity, copiousness and shareability, community-ization, and personalization are more distinctive, which, according to the theory of information asymmetry, assist in reducing the information asymmetry of listed companies. Researchers have also conducted studies on new media coverage [16]. Takeda and Wakao (2014) proposed that the related coverage of a company by new media such as Twitter would have an impact on the company’s stock trading, asset pricing [17]. Sun et al. (2018) found that listed companies’ microblogs and relatively active social media platforms positively influence the risk reduction of share price crashes [18]. Using data from 102 companies with active WeChat subscription accounts, Wang and Pan (2017) found that WeChat disclosure of related information about a company can increase its stock trading volume and further suggested that the impact of new media disclosure on liquidity is more significant among smaller companies [19]. Zhao et al. (2017) argued that auditors not only pay attention to the original information in new media, but also its reproduction whereas the likelihood of auditors issuing non-standard auditing opinions is significantly and positively related to the number of negative reports published on new media [20]. Further research confirmed that owing to the current inadequacy of China’s new-media-related legal system, negative reports on new media would shorten the time limit for listed companies to be punished for violations to a certain extent, albeit not increasing the likelihood of them being punished by governmental regulators. This also indicates that the rapid development of new media has allowed the timely exposure of some irregularities, granting auditors more comprehensive access to information, and thereby reducing the information asymmetry of listed companies. Some studies have found that social media also has corporate governance effects. Wang et al. (2020) found that social media can promote the management to voluntarily disclose earnings forecasts and improve the enthusiasm for timely disclosure of bad news [21]. Ang et al. (2021) found that social media news release can prompt management to abandon acquisition proposals that reduce the value of the company [22]. Sun et al. (2020) found that social media has corporate governance effect and can inhibit the positive earnings management behavior of companies [18].

#### 2.1.3 Media reports and auditors’ professional judgment.

Media coverage plays an important role in conveying information to the public and can impact auditors’ professional judgments. Xiao and Zhang (2016) proposed that auditors tune their risk perceptions towards clients out of a sense of self-protection, which will result in accounting firms charging a higher risk premium to audit fees; that is, auditors’ risk assessment will increase with increased media attention [14]. However, scholars such as Ran et al. (2016) suggested that civil litigation could bring direct losses, such as compensation to accounting firms, along with indirect losses, such as destruction of reputation, on which accounting firms rely more heavily than companies in other industries [23]. This is mainly because an accounting firm’s reputation is an intangible asset formed through its auditing activities with high costs over a long period, whereas listed companies use important indicators to choose their auditing firms. Thus, accounting firms incur a relatively high cost to repair reputational damage. The accounting industry is highly competitive and large firms with good reputations charge audited companies significantly higher audit fees because of the reputation incentive effect. In 2006, the implementation of China’s new securities law and the first case of an accounting firm held liable for financial fraud by an audited company converted the potential risk of bearing civil liability for misrepresentation into a realistic and predictable litigation risk. By the end of 2013, all of China’s securities-qualified accounting firms had completed their transformation to a Special General Partnership (SGP) form of organization. The conversion of accounting firm partners’ original limited liability into today’s unlimited liability means that partners’ exposure to legal risks, such as civil litigation risk, has increased significantly, which, in turn, suggests that the risk premium component of audit fees will also rise.

Thus, media attention and coverage may have a commensurate impact on a company by exposing a range of problems in it. Auditors’ attention has certainly been drawn to media coverage that has factored into the auditing procedure, whereby audit opinions and fees are ascertained in light of the risk premium and working resources invested. More importantly, new media spread faster and wider than traditional paper media, which may have impacted audit fees more directly. Thus, the following hypotheses were made:

Hypothesis 1: Audit fees increase when listed companies receive negative new media coverage.

If the positive relationship between a company’s negative new media coverage and its audit fees can be verified, then the causes and pathways leading to increased audit fees would be worthy of more in-depth analysis. The pricing criteria for accounting firms’ audit fees are inextricably linked to the breakdown of audit fees, which is two-fold: basic costs and risk premiums. Li et al. (2020) points out that the main basis for determining audit fees is the level of human resources invested by a firm and the audit risk it must bear. Risk premium theory provides a further explanation for the increase in audit fees [24]. When a listed company experiences negative media coverage, the accounting firm will heighten its assessment of auditing costs when deciding audit fees. For an auditor, when the to-be-audited company has negative media coverage, two strategies will be adopted to respond to the risk signals released. First, for companies with negative media coverage, the auditor will increase the level of effort, perform the audit procedures with caution and care, and devote more auditing efforts, resulting in higher audit costs and thus higher audit fees. Second, drawing on insurance theory for auditing demand, the auditor, upon learning that the company has negative media coverage, that is, knowing that a greater audit risk will be assumed, will consider the risk premium when assessing audit fees and charge higher audit fees as a response and compensation against future losses from audit failure. Therefore, in performing auditing procedures, how does an auditor choose to increase the level of effort or simply charge a risk premium, thereby increasing audit fees to cope with the risk? Based on this, we proposed Competing Hypothesis 2.

Hypothesis 2a: Negative new media coverage of listed companies in China is positively correlated with the audit time lag.

Hypothesis 2b: Negative new media coverage of listed domestic companies has no impact on the audit time lag.

### 2.2 Impact of the relationship between analyst tracking and audit fees

For investors in the securities market, the management of listed companies may take advantage of information asymmetry to earn excess returns, which, in turn, would increase investors’ investment risks and costs, thereby underscoring the important role of analysts. For listed companies, analyst tracking not only affects the mechanism of corporate governance and decision-making but also lessens the degree of information asymmetry between listed companies and investors. Yu and Tian (2011) studied corporate governance mechanisms under media spotlight using analyst tracking as a control variable [25]. The study showed that a large amount of media attention puts immense market pressure on managers, forcing them to engage in accrual-based surplus management to meet market expectations, which is more pronounced in situations where the number of analysts and the proportion of shares held by institutional investors are high.

On the one hand, with analyst tracking as a form of external monitoring for listed companies, the more attention they receive, the more reputational pressure it will create for auditors, who must work hard to uncover as much risk as possible. Xing and Chen (2013) argued that the auditor, in confirming audit fees, must aptly assess the corporate governance of the audited entity and its environment, and dig deeper into the business risks behind it to assess the risk of material misstatement during the audit [26]. If the assessed risk of material misstatement is high, then the auditor needs to perform special auditing procedures to reduce the risk of audit failure. Both audit risk and audit input have an impact on the recognition of audit fees in this process. To a certain extent, the higher the number of analyst trackers following a company, the more attention investors pay to the listed company. In such an environment, a failed audit has a detrimental effect on its reputation and that of the accounting firm involved, while concurrently increasing the likelihood of investors complaining to the authorities. Consequently, auditors are more vigilant in the event of negative media coverage of a listed company by adhering to prudent working principles and increasing their own risk premiums.

Han et al. (2021) believed that analysts have the ability and conditions to understand the company’s operation, and can reflect the information disclosure behavior inconsistent with the company’s actual situation into their research reports or earnings forecasts and stock valuations, so as to improve the management’s estimation of future reputation risk [27]. On the other hand, analyst tracking plays the role of rapidly diffusing and deeply mining information. Analysts can capture the most valuable information on listed companies with their business expertise and conduct an in-depth analysis by combining their rich background knowledge of an industry. More importantly, analyst tracking assists in enhancing market efficiency, transferring information, and strengthening listed companies’ knowledge. Therefore, once problems found by the analysis and research on listed companies are passed on to the accounting firm, the auditor can put in the corresponding audit force to discover and verify the problems as early as possible, thereby reducing the audit risks that may occur. In the event of a listed company’s negative media coverage, the auditor will inevitably devote more effort or perform special auditing procedures to confirm and clarify the risk factors of the listed company as a result of the analysts’ follow-up.

In summary, considering the workings formalities of the formation of reputation-based pressure mechanisms, auditors will be influenced by analysts’ behavior when conducting initial business activities, while public attention will be higher for listed companies with more analyst tracking, where not only auditors but also accounting firms face higher audit risk in the case of audit failure. Therefore, in the case of companies being under negative media coverage, analysts will focus and amplify the negative coverage of the listed company and the accounting firm will also face higher audit risk which in turn will increase audit fees. Accordingly, we propose Hypothesis 3 as follows:

Hypothesis 3: Analyst-tracking increases the impact of negative new media coverage on audit fees.

**Fig 1:**
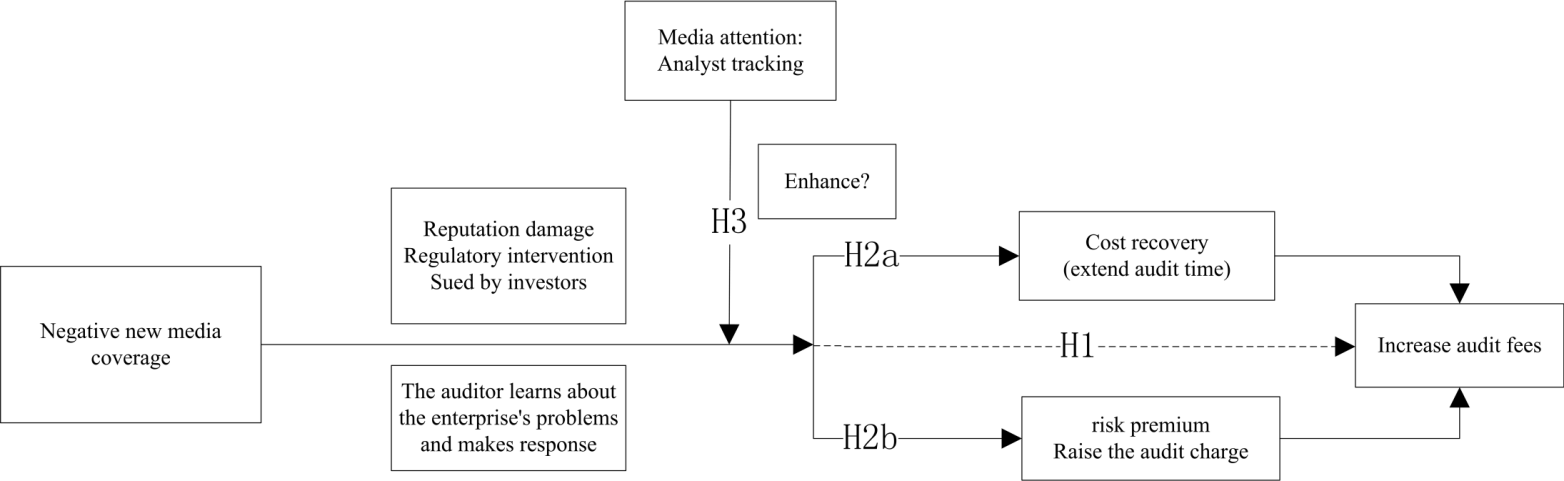
The transmission mechanism between negative new media coverage and audit fees.

To clearly demonstrate the logical relationship between these issues, Fig 1 analyzes, in detail, the transmission mechanism of negative new media coverage of audit fees.

## 3. Sample collection and data sources

### 3.1 Sample collection

The sample period of this paper is 2017-2019. The sample period started in 2017 because, in March, 2016, the Chinese government issued a resolution on the 13th five-year plan for national economic and social development, and dedicated the chapter “expanding cyberspace”, proposing eight major informatization projects such as the “Internet +” action and network security assurance, to promote the strategy of strengthening the country through network. Wechat official account has been widely used by wechat users since 2017. Referring to the research of Dai et al. (2011), it is generally believed that China Securities Journal, Securities Daily, Securities Times, Shanghai Securities News, China Business News, Economic Observer, 21st Century Business Herald, and China Business News are the mainstream financial newspapers in China [28]. Since 2017, the eight official wechat official accounts founded by the most influential national financial newspaper, namely, China Securities Journal, Securities Daily, Securities Times, Shanghai Securities News, Shanghai Securities News, China Business News, Economic Observer, 21st Century Business Herald, and China Business News, have become key positions for new media.

The deadline for the sample is 2019. Due to the impact of the COVID-19 epidemic in early 2020, a large amount of auditing work was done online for that year, and audit fees were significantly different from previous years. Although the sample period of this paper is 2017-2019, China’s economy is no longer affected by the epidemic. The sample used in this paper can still provide empirical experience for today’s Chinese enterprises. This paper takes the non-financial industry listed companies on the main board of A-share in Shanghai Stock Exchange from 2017 to 2019 as samples, and processes the sample as follows: ① excludes ST, * ST, S * ST companies, as the data on these companies may be unjustified; ② excludes listed companies with missing main variables as missing data may impact the reliability of the results. After the data screening process described above, 3620 observations were obtained, of which 1162 observations were for 2017, 1221 for 2018 and 1237 for 2019.

### 3.2 Data sources

The data in this study are mainly sourced from the CSMAR database, the WIND database, the website of the Chinese Institute of Certified Public Accountants, the official WeChat public websites of the China Securities Journal, Voice of Securities Daily, Securities Times, China Business Journal, The Economic Observer, 21st Century Business Herald, and China Business Network, which are the seven most influential nationwide financial newspapers. The rapid development of digital technology has significantly improved the timeliness of new media information propagation. WeChat is a new media platform that has an extensive influence on the general public’s daily lives and work. Compared to traditional media, new media have lower barriers to entry and are easier to operate which only require a simple application for registration to operate the “media of your own.” However, because of the time lag in legislation, laws and regulations are still largely absent from the self-media sector in China, and many self-media are operated by individuals who circulate information of varying quality. The study by Liu et al. (2017) used eight of the most influential nationwide financial newspapers: China Securities Journal, Securities Daily, Securities Times, Shanghai Securities News, China Business News, Economic Observer, 21st Century Business Herald, and China Business News—as data sources for official media reports [29].

Therefore, drawing on this practice against the backdrop of the current rapid development of new media, this study uses the official WeChat public accounts of the China Securities Journal, Voice of Securities Daily, Securities Times, Shanghai Securities News, China Business Journal, The Economic Observer, 21st Century Business Herald, and China Business Network as data sources for negative new media reports of listed companies in China. A web crawler was utilized to obtain historical information on the eight official public accounts above, whereas the number of occurrences of negative terms was documented by matching a list of negative keywords with the article titles and contents therein, with reference to the study by Dai et al. (2013) [30]. A total of 59,772 historical messages of the eight public accounts from January 1,2017, to December 30, 2019, were obtained using the crawler, of which 5,083 were confirmed as negative reports after keyword matching and manual checking. Furthermore, 2,743 negative reports covering 687 enterprises were retained in the SSE Composite Index after pertinent elimination.

## 4 Definition of variables and model construction

### 4.1 Dependent variables

#### 4.1.1 Audit Fees (LNFEE).

Drawing on the study by Xiao and Zhang (2016) [14], the natural logarithm of the total Audit Fees paid by the audited entities was used as the dependent variable, Audit Fees (LNFEE).

#### 4.1.2 Audit Time Lag (LNARL).

To study the mechanism by which negative media coverage impacts audit fees, the Audit Time Lag variable was introduced. Audit Time Lag is an indicator of the time it takes for an auditor to perform the auditing work and indirectly reflects the auditor’s effort in performing the auditing work [31]. This study uses the natural logarithm of the number of calendar days plus one between the balance sheet date and the audit report date as the dependent variable, Audit Time Lag (LNARL).

### 4.2 Explanatory variables

#### 4.2.1 Negative New Media Coverage (MEDIA).

**Fig 2:**
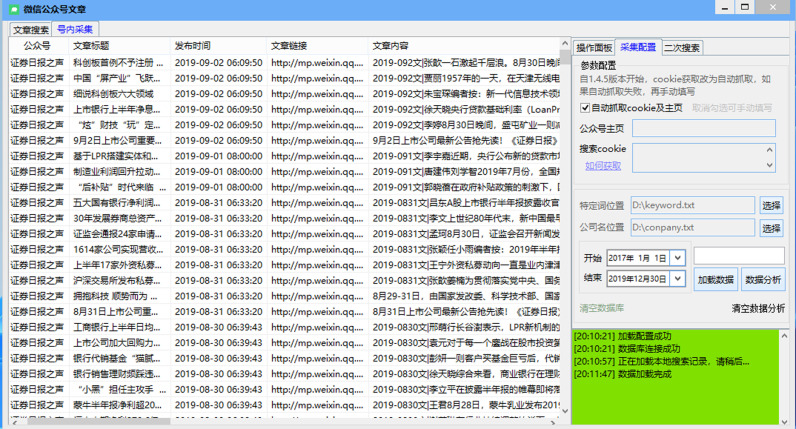
Crawler interface for collecting variable data of new media negative reports (MEDIA).

This study uses the “number of negative new media reports” as a measure of the explanatory variable Negative New Media Coverage (MEDIA). Combined with the era background of the rapid development of new media, referring to the research of Dai et al. (2011), this study uses the official WeChat public accounts of the China Securities Journal, Voice of Securities Daily, Securities Times, Shanghai Securities News, China Business Journal, The Economic Observer, 21st Century Business Herald, and China Business Network as data sources for negative new media reports of listed companies in China. A web crawler was utilized to obtain historical information on the eight official public accounts above, whereas the number of occurrences of negative terms was documented by matching a list of negative keywords with the article titles and contents therein, with reference to the study by Dai et al. (2013) [30]. The crawler interface used in this study to collect the variable data of new media negative reporting (MEDIA) is shown in (Fig 2).

The crawler was used to obtain a total of 59772 historical messages from eight public accounts from January 1, 2017 to December 30, 2019. After matching keywords and manual verification, there were 5083 negative reports. After relevant elimination of enterprises, 2743 negative reports were related to the enterprises of the Shanghai Stock Exchange Index, involving 687 Enterprises.

#### 4.2.2 Analyst Tracking (ANATRACK).

The number of analysts at the audited entity.

### 4.3 Control variables

This paper draws lessons from the existing relevant research on auditing and media reporting, and the control variables used include two levels: corporate characteristics and audit work characteristics. Among them, the company’s characteristics are measured by variables such as the company’s asset liability ratio (LEV), the company’s operating risk (CURRENT), the company’s litigation risk (ADDRESS), the nature of the enterprise (NATURE), the degree of equity concentration (CR1), and the proportion of executives’ shareholding (INS). The characteristic variables of audit work are selected to measure such variables as audit opinion type (OPINION), firm reputation (BIG4), and audit workload (SALES). The specific variables are defined as follows:

Debt-to-Assets Ratio (LEV): The ratio of total liabilities at the end of the period divided by total assets at the end of the period.
Type of auditor opinion (OPINION): 1 if the auditor’s opinion of the audited entity is unqualified and 0 if it is not.
Corporate Operation Risk (CURRENT): The ratio of total current assets at the end of the period divided by total current liabilities at the end of the period.
Corporate Litigation Risk (ADDRESS): A value of 1 if the unit is domiciled in Beijing, Shanghai, Tianjin, Guangdong, or Zhejiang and 0 otherwise.
Firm Reputation (BIG4): 1 if Big Four, 0 otherwise.
Audit Workload (SALES): The natural logarithm of the auditing entity’s operating income.
Nature of Business: 1 for state-owned enterprises, 0 otherwise.
Concentration of Shareholding (CR1): The sum of the shareholdings of the top three largest shareholders of the audited entity.
Company Size of the Audited Entity (SIZE): The natural logarithm of the audited entity’s total assets at the end of the period.
Executives Shareholding (INS): The ratio of the number of shares held by executives of the audited entity to the number of capital shares of the company.

### 4.4 Model setup

To test Hypothesis 1–that is, the relationship between Negative New Media Coverage of the audited entity and Audit Fees–this study builds a multiple linear regression Model 1.


$$\begin{array}{l} LNFEE=\beta_{0}+\beta_{1}MEDIA+\beta_{2}LEV+\beta_{3}OPINION+\beta_{4}CURRENT+\beta_{5}ADDRESS+\beta_{6}BIG4+\beta_{7}SALES\\ +\beta_{8}NATURE+\beta_{9}CR1+\beta_{10}SIZE+\beta_{11}INS+\beta_{12}\sum INDi+\beta_{13}\sum Yeart+\varepsilon \end{array}$$

If$\beta_{1}$ is significantly greater than 0 across the board, this would indicate a positive relationship between negative new media coverage and audit fees, that is, Hypothesis 1 holds; conversely, Hypothesis 1 does not hold.

To test Hypothesis 2, that is, the relationship between Negative New media coverage of the audited entity and the Audit Time Lag, multiple linear regression model 2 was built.


$$\begin{array}{l} LNARL=\beta_{0}+\beta_{1}MEDIA+\beta_{2}LEV+\beta_{3}OPINION+\beta_{4}CURRENT+\beta_{5}ADDRESS+\beta_{6}BIG4+\beta_{7}SALES\\ +\beta_{8}NATURE+\beta_{9}CR1+\beta_{10}SIZE+\beta_{11}INS+\beta_{12}\sum INDi+\beta_{13}\sum Yeart+\varepsilon \end{array}$$

If$\beta_{1}$ is significantly greater than 0 in all cases, this would indicate that Negative New Media Coverage and Audit Time Lag are positively correlated, that is, Hypothesis 2 holds; conversely, Hypothesis 2 does not hold.

In order to test hypothesis 3, that is, whether Analyst Tracking would intensify the effects of Negative New Media Coverage on Audit Fees, in this paper, the sample is subdivided into Group 1 being the firms where the number of analysts tracking organizations more than median and Group 1 being the firms where the number of analysts tracking organizations fewer than median and model 3 was built as follows:


$$\begin{array}{l} LNFEE=\beta_{0}+\beta_{1}MEDIA+\beta_{2}LEV+\beta_{3}OPINION+\beta_{4}CURRENT+\beta_{5}ADDRESS+\beta_{6}BIG4+\beta_{7}SALES\\ +\beta_{8}NATURE+\beta_{9}CR1+\beta_{10}SIZE+\beta_{11}INS+\beta_{12}\sum INDi+\beta_{13}\sum Yeart+\varepsilon \end{array}$$

## 5. Empirical results and analysis

### 5.1 Descriptive statistics

From the descriptive statistics of the variables in Table 1, it can be seen that the mean of Audit Fees (LNFEE) is 13.8777 and the median is 13.7102 whereas the mean and median of negative media coverage MEDIA are 0.3908 and 0 respectively; the mean of BIG4 is 0.0908, which indicates that listed companies in China are more willing to choose local accounting firms for auditing; the mean of NATURE is 0.2023 while the maximum of the shareholding ratio of the top three major shareholders is 96% with the minimum being 10% and the mean being 51.74%.

**Table 1:** Descriptive statistics of the sample.

Indicators	No. Obs.	Minimum	Average	Median	Maximum	Standard deviation
**LNFEE**	3620	12.0436	13.8777	13.7102	17.9769	0.7691
**LNARL**	3620	3.8918	4.6658	4.7274	4.8040	0.1463
**MEDIA**	3620	0.0000	0.3908	0.0000	11.0000	0.8564
**ANATRACK**	3620	0	20.4170	13	85	18.9499
**LEV**	3620	0.0084	0.4423	0.4344	0.9952	0.2011
**OPINION**	3620	0.0000	0.9522	1.0000	1.0000	0.2133
**CURRENT**	3620	0.1481	2.3638	1.6062	80.6637	3.2956
**ADDRESS**	3620	0.0000	0.4494	0.0000	1.0000	0.4975
**BIG4**	3620	0.0000	0.0980	0.0000	1.0000	0.3458
**SALES**	3620	16.2228	22.0389	21.8563	28.6927	1.5773
**NATURE**	3620	0.0000	0.2023	0.0000	1.0000	0.4018
**CR1**	3620	0.1009	0.5174	0.5266	0.9604	0.1738
**SIZE**	3620	18.4908	22.7113	22.4931	28.5200	1.4615
**INS**	3620	0.0000	5.1169	0.0065	77.8689	12.3099

### 5.2 Multiple regression analysis

From the multiple regression results in Column 1, Table 2, it can be found that Negative New Media Coverage (MEDIA) is positively correlated to Audit Fees (LNFEE), and the estimate on MEDIA is statistically significant at 1% significance level, which may indicate that the more negative new media coverage, the more auditors will pay attention to such coverage and thereby increase audit fees as risk premiums or take more steps in auditing procedures by increasing audit efforts, which will in turn increase audit fees. This finding suggests that negative media coverage has a direct impact on a company’s audit fees. Thus, hypothesis 1 is verified. On the one hand, as an information medium, new media has the function of information transmission (Zou, et al. 2019) [32]. Compared with general media reports, negative media reports are often more likely to attract widespread public attention, which makes enterprises more vulnerable to administrative punishment by regulatory authorities and increases the company’s business, financial and litigation risks (Zhang and Yu, 2013) [33]. The audit demand insurance theory believes that audit is a risk transfer mechanism, and these risks from client companies will be borne by auditors to a certain extent, and the risk of auditors encountering litigation and civil compensation will increase accordingly (Yu, et al. 2013) [34]. The more negative media reports, the higher the risk level of the enterprise, which makes auditors face more potential litigation and reputation loss risks (Hong et al., 2021) [35]. Therefore, auditors will evaluate their potential risks and losses according to the negative reports of the media on customers, and take corresponding countermeasures to reduce risks and possible losses, such as investing more time and energy, which will lead to the increase of audit fees. Previous studies have focused on the relationship between enterprise risk and audit quality from the perspective of negative media reports on enterprise operation and litigation risk. The text combines the research in the above fields through the negative reports of new media and audit fees, indicating that the negative reports of media will eventually be transmitted to the improvement of enterprise audit fees, enriching the research in related fields.

**Table 2:** Regression results for model 1, 2 and 3.

Variables	Adverse publicity and audit fees	Adverse publicity and audit delays	The moderating role of analyst tracking	
	LNFEE (audit fees)	LNARL (audit time lag)	More anatracks	Fewer ANATRACKS
			LNFEE (audit fees)	LNFEE (audit fees)
**MEDIA**	0.070^***^	0.000295	0.0909^***^	0.00597
	(3.664)	(0.00296)	(0.0217)	(0.0247)
**LEV**	0.013	0.0887^***^	0.0522	-0.0731
	(0.248)	(0.0176)	(0.0658)	(0.0815)
**OPINION**	-0.188^***^	0.0290^**^	-0.199^**^	-0.115^***^
	(-4.616)	(0.0115)	(0.0794)	(0.0431)
**CURRENT**	0.006^***^	0.000715	0.00703	0.00279
	(2.759)	(0.000840)	(0.00442)	(0.00232)
**ADDRESS**	0.210^***^	-0.0199^***^	0.219^***^	0.125^***^
	(13.021)	(0.00546)	(0.0182)	(0.0233)
**BIG4**	-0.160^***^	-0.00125	-0.163^***^	-0.0718^**^
	(-7.657)	(0.00657)	(0.0228)	(0.0313)
**SALES**	0.110^***^	-0.00680	0.115^***^	0.106^***^
	(7.971)	(0.00495)	(0.0148)	(0.0201)
**NATURE**	0.039^*^	0.00840	0.0120	0.105^***^
	(1.954)	(0.00609)	(0.0219)	(0.0345)
**CR1**	0.253^***^	-0.0487^***^	0.402^***^	0.0497
	(5.004)	(0.0167)	(0.0597)	(0.0725)
**SIZE**	0.322^***^	0.000670	0.330^***^	0.260^***^
	(20.032)	(0.00538)	(0.0175)	(0.0265)
**INS**	0.000	5.95e-05	-0.000101	0.000370
	(0.260)	(0.000201)	(0.000662)	(0.000775)
**INDUSTRY**	Control	Control	Control	Control
**YEAR**	Control	Control	Control	Control
**Constant**	4.114^***^	4.759^***^	3.737^***^	5.530^***^
	(21.764)	(0.0494)	(0.233)	(0.356)
**Observations**	3,620	3,620	2408	1,212
**R-squared**	0.684	0.100	0.691	0.592

Note: Standard deviations are reported in parentheses; *, **, and *** indicate significance at the 10%, 5%, and 1% statistical levels respectively, as in subsequent tables.

From the multiple regression results in Column 2 and Table 2, it can be seen that there is no correlation between Negative New Media Coverage MEDIA and Audit Time Lag LNARL, implying that the more negative the new media coverage, the more auditors will pay attention to such coverage, thereby raising audit fees to absorb the risk premium rather than committing more time and effort to audit work. Therefore, the transmission of negative new media coverage of higher audit fees occurs via a risk premium.

From the results of the multiple regressions in Column 3 vs. Column 4 of Table 2, it is not difficult to find that the explanatory variable Negative New Media Coverage (MEDIA) is also significantly and positively correlated with the dependent variable Audit Fees (LNFEE) only in the group of firms with more analyst tracking, whereas this correlation is found to be statistically insignificant for their peers with fewer analysts tracking. This result suggests that for firms under negative news coverage, audit fees are assessed as higher in the presence of analyst tracking, which further indicates that greater attention to a firm will lead auditors to respond more strongly to this potential downside risk in the form of higher risk premium charges.

## 6. Further research

### 6.1 The moderating role of the ownership nature of an enterprise

China’s enterprises are categorized into state-owned and non-state-owned enterprises, based on the nature of their ownership. We further explore how the impact of a negative new media coverage on audit fees differs according to the nature of ownership in China’s institutional context. Compared to non-state enterprises, state-owned enterprises are generally larger, and because of the uniqueness of their ownership, they tend to receive more policy and financial support from the government [36]. Wang et al. (2019) showed that the impact of market or analyst attention on audit fees varies among firms of different ownerships [37]. A study by Wei et al. (2021) showed that audit fees are significantly lower for SOEs(State-owned enterprise) than for non-SOEs, in case of companies with affiliated management [38]. Hence, is there a difference between these two types of firms with different natures of ownership when facing negative new media coverage? Will the audit fees assessed by accounting firms differ when dealing with enterprises with different ownership characteristics? In this study, the companies are grouped into subsamples in accordance with their nature of ownership with Group one being state-owned enterprises and Group two being their non-state-owned counterparts. The aforementioned questions are answered by rerunning the regression in Column 2 and Table 2, respectively, for each subgroup.

The regression results from Table 3 show that, in case of companies under negative new media coverage, non-SOEs are more likely to have their audit fees raised by accounting firms relative to SOEs. Compared to non-SOEs, SOEs engage higher market attentions which leads to a relatively sound system and clearly defined standard of information disclosure. Thus, accounting firms and CPAs have relatively small discrepancies in audit fees due to information asymmetry when taking over their audit work. In addition, Zhou and Yao (2015) believes that state-owned enterprises are largely subject to government intervention, which will weaken the external governance effect of the media [39].

**Table 3:** Multiple regression results for the moderating effect of the nature of property rights.

Variables	State-owned enterprises	Non-state enterprises
	LNFEE (audit fees)	LNFEE (audit fees)
**MEDIA**	0.00792	0.0500^***^
	(0.0130)	(0.0107)
**Controls**	Control	Control
**INDUSTRY**	Control	Control
**YEAR**	Control	Control
**Observations**	732	2,888
**R-squared**	0.816	0.695

The conclusion of this paper is mutually corroborated with the above research results, further proving that the attention and negative reports of the news media have a higher effect on reducing information asymmetry in non-state-owned enterprises than in state-owned enterprises. For non-SOEs, media attention and conveyed negative information would render CPAs more sensitive to audit risks, thereby charging a commensurately higher risk premium and cost compensation. Therefore, from the perspective of information effectiveness, accounting firms are more responsive to media reports on non-SOEs than on SOEs.

### 6.2 The moderating role of the size of accounting firms

DeAngelo (1981) pointed out that large accounting firms, especially the international “Big Four”are more likely to find material misstatements in financial statements because of their experience and capability to identify and respond to risks [40]. Pan (2011) pointed out that the international “Big Four” tend to hedge high-risk venture risks by issuing strict audit opinions rather than charging higher risk premiums [41]. In addition, Qi et al. (2004) pointed out that large firms invest a large amount of money in creating and maintaining their brands and reputations because of the great importance they attach to brand building and maintenance [42]. Therefore, do accounting firms of different sizes react differently to possible risks when learning about negative new media coverage of audited companies? For comparison, the sample was subdivided into Group one: (Big Four accounting firms, and Group two: (non-Big Four accounting firms).

The regression results in Table 4 show that non-Big Four accounting firms are more likely to assess higher audit fees than Big Four accounting firms in response to potential audit risks in the event of negative media coverage of listed companies. This indicates that Big Four accounting firms do not increase their audit fees to address audit risk because of their capability to identify and respond to the risks associated with negative media coverage of their clients, whereas non-Big Four accounting firms do so. This also indirectly shows that accounting firms of larger scale have more standardized management and more confidence in their professional capabilities thereby never increasing their audit pricing randomly because of client issues. In contrast, smaller accounting firms raise their audit fees to cope with risk, given their relatively chaotic internal management and fierce competition.

**Table 4:** Multiple regression results for the moderating effect of accounting firm size.

Variables	Non-Big Four	The Big Four
	LNFEE (audit fees)	LNFEE (audit fees)
**MEDIA**	0.0523^***^	0.0224
	(-0.0108)	(-0.0399)
**Controls**	Control	Control
**INDUSTRY**	Control	Control
**YEAR**	Control	Control
**Observations**	2,621	267
**R-squared**	0.562	0.869

### 6.3 The relationship between the specified consequences of negative new media coverage and audit fees

Impact of negative new media coverage on audit fees and its transmission mechanism have been studied with analyst tracking introduced as a moderating variable, as well as the role and effect of the nature of business and the size of accounting firms. Other studies contend that varying degrees of negative media coverage may have different effects on audit fees. Liu et al. (2014) pointed out that the impact of negative accounting coverage on auditors differs from that of other types [12]; Mi et al. (2019) studied the impact of non-punitive regulatory instruments on audit fees, finding that listed companies that received inquiry letters from the Stock Exchange had significantly higher audit fees than those that did not [15]. Thus, from another perspective, this study further investigates different categories of negative reports based on their specific consequences: rumors, regulatory inquiries, and punishment.

Rumor (MEDIAONLY): WeChat public accounts published negative remarks but did not receive inquiry letters or penalties.
Regulatory Inquiry (INQUERY): Companies, in addition to incurring negative comments on WeChat public accounts, received inquiry letters from the SFC (Source: Shanghai Stock Exchange official platform. http://www.sse.com.cn//disclosure/credibility/supervision/inquiries/)
Punishments (PUNISH): Companies or executives received penalties or sanctions for negative comments on WeChat public accounts. (Source: China Research Data Service Platform (CNRDS))

Hypotheses 1–4 would be tested separately, and the results are presented in Tables 5 and 6. Multiple regression results state that Negative New Media Coverage (MEDIA), provided that it is in general positively correlated with Audit Fees (LNFEE), is still positively correlated with audit fees across the three types— rumors, regulatory inquiries, and punishment—with estimates being statistically significant at 1% significance level. However, none are statistically significant with Audit Time Lag as a dependent variable, which indicates that auditors pay attention to negative new media coverage and thereby increase audit fees in response to higher risk premiums rather than the extension of audit time lag.

**Table 5:** Multiple regression results for testing hypothesis 1 and hypothesis 2 by group.

Variables	Adverse publicity and audit fees			Adverse publicity and audit time lag		
	LNFEE	LNFEE	LNFEE	LNARL	LNARL	LNARL
**MEDIAONLY**	0.0660^***^			-0.000539		
	(0.0203)			(0.00289)		
**INQUERY**		0.0472^***^			0.00243	
		(0.0117)			(0.00392)	
**PUNISH**			0.0471^***^			-0.00151
			(0.0126)			(0.00574)
**Controls**	Control	Control	Control	Control	Control	Control
**Ind**	Control	Control	Control	Control	Control	Control
**Year**	Control	Control	Control	Control	Control	Control
**Observations**	3,620	3,620	3,620	3,620	3,620	3,620
**R-squared**	0.710	0.710	0.710	0.125	0.125	0.125

**Table 6:** Multiple regression results for group testing hypothesis 3.

The moderating role of analyst tracking						
No. of Analyst Tracking (whether more than median)	Yes	No	Yes	No	Yes	No
**VARIABLES**	LNFEE	LNFEE	LNFEE	LNFEE	LNFEE	LNFEE
**MEDIAONLY**	0.0660^***^			-0.000539		
	(0.0203)			(0.00289)		
**INQUERY**		0.0472^***^			0.00243	
		(0.0117)			(0.00392)	
**PUNISH**			0.0471***			-0.00151
			(0.0126)			(0.00574)
**Controls**	Control	Control	Control	Control	Control	Control
**Ind**	Control	Control	Control	Control	Control	Control
**Year**	Control	Control	Control	Control	Control	Control
**Observations**	3,620	3,620	3,620	3,620	3,620	3,620
**R-squared**	0.710	0.710	0.710	0.125	0.125	0.125

With MEDIAONLY, INQUERY, and PUNISH all being positively correlated with LNFEE as described above, the results of using Analyst Tracking as a group variable show that for a company with negative coverage, analyst tracking is always a factor that the company’s auditor considers when assessing audit fees; moreover, the higher the number of analyst tracking, the higher the audit fees. This indirectly implies that the more attention a business receives, the more intensely its auditor responds to potential risks, thereby generating a certain risk premium.

## 7. Robustness check

### 7.1 One period lag

This study reruns the regression analysis using Negative New Media Coverage (MEDIA) from the previous period as a proxy for the key independent variables. The results in Table 7 show that negative new media coverage (before and after categorization) and audit fees have a positive relationship.

**Table 7:** Robustness tests for the main regression.

VARIABLES	LNFEE	LNFEE	LNFEE	LNFEE
**MEDIA**	0.0418^***^			
	(0.0107)			
**MEDIAONLY**		0.0475^**^		
		(0.0222)		
**INQUERY**			0.0614^***^	
			(0.0155)	
**PUNISH**				0.0490^***^
				(0.0182)
**Controls**	Control	Control	Control	Control
**Ind**	Control	Control	Control	Control
**Year**	Control	Control	Control	Control

### 7.2 Substitution of variables

Replacement of the explanatory variables with Negative New Media Coverage (MEDIA, MEDIAONLY, INQUERY, PUNISH):

For the measure of negative media coverage, in the previous regression analysis, we have adopted the “number of negative media coverage” as the measure of negative media coverage (MEDIA, MEDIAONLY, INQUERY, PUNISH). Therefore, in this robustness check, the explanatory variables MEDIA, MEDIAONLY, INQUERY, and PUNISH are all set as dummy variables; that is, if a listed company experiences negative new media coverage, then MEDIA, MEDIAONLY, INQUERY, and PUNISH are assigned a value of 1, and 0 otherwise. Finally, once we substituted the calculated alternative variable measures into the original regression model 1, Hypothesis 1 was validated. The results are shown in Table 8.

**Table 8:** Robustness tests for the main regression.

Variables	LNFEE	LNFEE	LNFEE	LNFEE
**MEDIA**	0.0662^***^			
	(0.0158)			
**MEDIAONLY**		0.0477^**^		
		(0.0210)		
**INQUERY**			0.0574^***^	
			(0.0191)	
**PUNISH**				0.0644^**^
				(0.0251)
**Controls**	Control	Control	Control	Control
**Ind**	Control	Control	Control	Control
**Year**	Control	Control	Control	Control

## 8. Conclusion

This study empirically finds that negative media coverage has a positive impact on auditors’ pricing and shows that there is a positive relationship between negative media coverage and a firm’s audit fees for the year, regardless of whether it is a rumor, regulatory inquiry, or penalty. However, negative media coverage has no positive relationship with the audit time lag for the year, suggesting that the mechanism via which negative media coverage is transmitted to audit fees is through a risk premium. In further research, it could be found that from the standpoint of enterprises, negative media coverage of non-SOEs was more likely to lead to higher audit fees while from the standpoint of accounting firms, “non-Big Four” firms were more likely to raise audit fees for ventures under negative media coverage. The moderating effect of analyst tracking clearly strengthens the impact of negative coverage on audit fees suggesting that auditors also considers the level of analyst attention while auditing firms. Meanwhile, companies with higher levels of attention are assessed higher audit fees in order to address risk and generate a risk premium. This study assists in clarifying the determining factors of audit fees and making full use of the monitoring role of new media to improve the information transparency of listed companies and boost the efficiency of resource allocation while providing a benchmark for the establishment of a feasible audit fee system.

The research of this paper has made contributions in at least the following three aspects: (1) It has enriched the research in the field of news media attention. Previous research has mainly focused on traditional media such as paper newspapers and government announcements. Now, new media has developed rapidly. Users take WeChat as the window to receive information from all parties in a timely manner. The impact on new media reports represented by WeChat official account should attract the attention of the academic community. This paper obtains the key variables of negative reports on WeChat official account by means of crawler, and it is of great theoretical and practical significance to study their impact on enterprise audit fees. (2) Enriched research in the field of audit fees. Most research related to corporate governance adopts public databases represented by CSMAR and WIND, or obtains data through manual collection, field research, and interviews. However, it is difficult to collect the key variables of the new media negative reports used in this article manually. This article uses a crawler to obtain the negative reports on WeChat official account by writing programs and software. Auditors, as important stakeholders of enterprises and users in the new media era, are connected to internal and external influences of auditors through the acquisition of this key variable. This breakthrough study investigates the impact of negative media coverage on enterprise audit costs. (3) The further classification of negative reports on new media in this article is an important innovation and enriches relevant research on media reporting. It has also made certain contributions to future scholars’ research on the impact of different types of negative reporting.

## Supporting information

S1 Table Data for all variables of this paper. (XLSX)
